# Progression of Ascending Aortic Dimensions in Masters Endurance Athletes

**DOI:** 10.1016/j.jacadv.2025.102163

**Published:** 2025-09-16

**Authors:** Nathaniel Moulson, Noah D. Boroditsky, Julien Wiese, Reid A. Mitchell, Mark J. Haykowsky, Stephen J. Foulkes, James Roberts, Saul Isserow, Barbara N. Morrison, James McKinney

**Affiliations:** a SportsCardiologyBC, The University of British Columbia Hospital, Vancouver, British Columbia, Canada; b Division of Cardiology, Faculty of Medicine, The University of British Columbia, Vancouver, British Columbia, Canada; c College of Health Sciences, Faculty of Nursing, University of Alberta, Edmonton, Alberta, Canada; d Hochgebirgsklinik Davos, Medicine Campus Davos, Davos, Switzerland; e Heart, Exercise and Research Trials Lab, St Vincent’s Institute of Medical Research, Fitzroy, Victoria, Australia; f Department of Radiology, The University of British Columbia, Vancouver, British Columbia, Canada; g School of Human Kinetics, Trinity Western University, Langley, British Columbia, Canada

**Keywords:** aneurysm, aorta, masters athletes, sports cardiology

**Figure:**
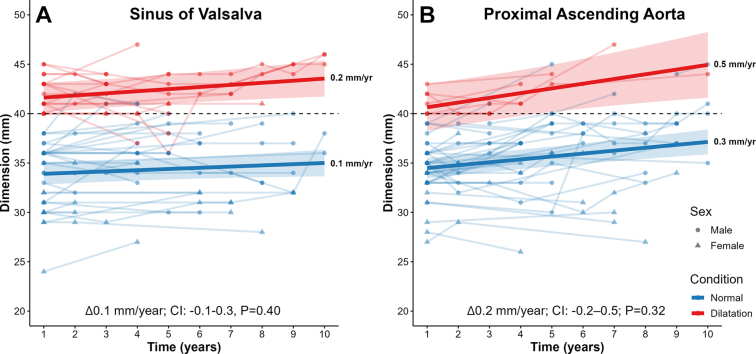

**What is the clinical question being addressed?**

What is the observed rate of change of ascending aortic dimensions of masters athletes with and without clinically significant baseline aortic dilatation?

**What is the main finding?**

The rate of aortic dimension progression was similar in masters athletes with and without baseline aortic dilatation.

Dilatation of the thoracic ascending aorta (≥40 mm) is a common finding in masters athletes (MAs), defined as individuals aged ≥35 years who engage in regular exercise training to improve fitness and/or participate in competitions.^1^ Amongst a cross-sectional cohort of elite male and female MAs (rowers and runners), 21% exhibited clinically significant aortic dilatation (AD).^1^ In young American football players, progression of aortic root size (0.6 mm/year) occurred over short-term follow-up (3 years)^2^; however, none of these athletes had clinically relevant AD. The natural history of change in aortic dimensions of MAs is unknown. Concern exists that increased hemodynamic load associated with continued high-volume exercise may accelerate AD.^3^ This study sought to determine the progression of AD in MAs who continued to exercise over a 10-year period.

A retrospective cohort analysis of MAs enrolled in the Masters Athlete Screening Study^4^ and subsequent 5-year follow-up was performed to evaluate progression of the sinus of Valsalva (SoV) and proximal ascending aorta (AAo) in participants with ≥2 clinically indicated transthoracic echocardiograms (TTEs). Aortic measurements were obtained from clinical TTE reports (single 2D measurement from leading edge to leading edge of aortic walls in diastole). Participants with a known aortopathy diagnosis, including bicuspid-associated aortopathy, were excluded. AD was defined as ≥40 mm for the SoV and/or AAo.^1^^,^^5^ Demographic, anthropometric, medical and exercise history, and clinical outcomes data were reviewed. After testing for normality, continuous variables are presented as mean ± SD. Fisher’s exact test was used to compare groups for categorical variables. Linear mixed-effects (LME) models were generated for the SoV and AAo. Estimated marginal means with 95% CI were calculated to obtain baseline dimensions. This study was approved by The University of British Columbia Research Ethics Board (H19-02554).

Fifty MAs (age 60 ± 8 years; 26% female; 45 ± 11 years active; 30 recreational, 12 competitive, 8 elite) met the inclusion criteria. Nineteen participants (38%) had AD (12 SoV, 2 AAo, and 5 both) and 31 (62%) had nondilated dimensions. Mean body mass index and body surface area were 25 ± 3 kg/m^2^ and 1.93 ± 0.25 m^2^, respectively. Average resting systolic and diastolic blood pressure were 136 ± 15 and 80 ± 7 mm Hg in the AD group compared to 125 ± 17 and 76 ± 8 mm Hg in the nondilated group. Hypertension was diagnosed in 46% of participants during the study period and was more common in the AD than nondilated group (68 vs 32%; *P* = 0.02). Metabolic equivalent of task (MET) volumes of all participants were 81 ± 39 (year 1), 91 ± 39 (year 5), and 72 ± 40 MET-h/week (year 10). There was no difference in baseline MET volumes between groups (*P* = 0.13). Exercise was predominately endurance based. Running (n = 21), cycling (n = 18), and hockey (n = 8) were most common. An average of 2 TTEs (IQR: 1) were performed at a mean interval of 5 ± 3 years. The estimated mean dimensions of the AD group were 42.1 mm (CI: 39.7-44.4) for the SoV and 40.2 mm (CI: 37.6-42.7) for the AAo vs 33.8 mm (CI: 32.6-34.9) and 34.2 mm (CI: 33.1-35.3) for the SoV and AAo of the nondilated group (Figure 1). The AD group SoV progression was 0.2 mm/year (CI: 0.1-0.4) compared to 0.1 mm/year (CI: -0.0 to 0.3) in the nondilated group. For the AAo, the AD group increased at 0.5 mm/year (CI: 0.1-0.8) vs 0.3 mm/year (CI: 0.2-0.4) in the nondilated group. There was no significant interaction between the time and aortic diameter of the SoV (Δ0.1 mm/year; CI: -0.1 to 0.3; *P* = 0.40) or AAo (Δ0.2 mm/year; CI: -0.2 to 0.5; *P* = 0.32) between AD and nondilated groups. Six (20%) of the nondilated group progressed to ≥40 mm. No participant in the AD group progressed to ≥50 mm.^3^

**Figure 1:**
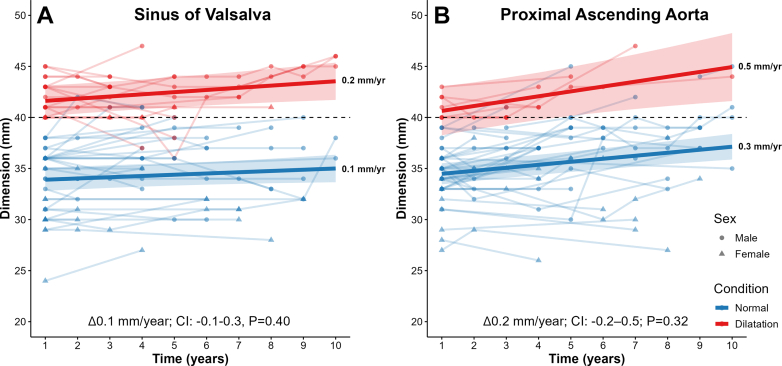
**Rate of Change in Aortic Dimensions of Aortic Dilation vs Nondilated Masters Athletes** Linear mixed-effects (LME) models (thick lines with CI shading) of the change in (A) sinus of Valsalva and (B) proximal ascending aorta dimensions over time between aortic dilatation (red) and nondilated (blue) groups. Thin lines represent individual male (circle) and female (triangle) measurements from transthoracic echocardiogram reports. Thick lines represent the estimates of the LME models. Colored shading represents 95% CIs of the LME model estimates. The dashed black line indicates the threshold for aortic dilatation (≥40 mm).

The major new findings of this study include the following: 1) no significant difference in the rate of change in aortic dimensions between the AD and nondilated group; 2) the observed rate of change for MAs with AD was 0.2 mm/year (CI: 0.1-0.4) at the SoV level and 0.5 mm/year (CI: 0.1-0.8) for the AAo, which is well below clinically significant rates of AD; and 3) hypertension was more common in MAs with AD.

This study provides important new insight into the rate of change of aortic dimensions in MAs who continued high-volume exercise. In nonathletes with ascending AD, expansion has been reported at 0.1 mm/yr.^5^ The observed rate of expansion in the present study is higher in both the dilated and nondilated groups, suggesting a potential impact of exercise on the expansion rate. However, these expansion rates are significantly below the 3 mm/yr expansion rate considered “rapid” growth.^5^ This suggests continued exercise training with clinical observation may be a reasonable strategy in MAs with AD.

Caution is required in extrapolating these findings to other populations, including young (<35 years) athletes and those with known aortopathies. Limitations include the chart review of clinical TTE reported measurements without central adjudication, predominantly male cohort, and absence of nonathlete controls. Further study is required to systematically evaluate the relationship between high-volume exercise, aortic dimensions, and associated clinical outcomes.

## Funding support and author disclosures

This work was supported by the Vancouver General Hospital and 10.13039/501100005247University of British Columbia Hospital Foundation, MITACs to Dr Morrison the 10.13039/501100000024Canadian Institute of Health Research (grant number: 157930) to Dr Morrison The authors have reported that they have no relationships relevant to the contents of this paper to disclose.
